# Effects of Choice Restriction on Accuracy and User Experience in an Internet-Based Geopolitical Forecasting Task

**DOI:** 10.3389/fpsyg.2021.662279

**Published:** 2021-07-14

**Authors:** Colin L. Widmer, Amy Summerville, Ion Juvina, Brandon S. Minnery

**Affiliations:** ^1^Kairos Research, Dayton, OH, United States; ^2^ASTECCA Laboratory, Department of Psychology, Wright State University, Dayton, OH, United States

**Keywords:** geopolitical forecasting, wisdom of crowds, workload, choice restriction, intelligent load distribution

## Abstract

Large-scale geopolitical forecasting tournaments have emerged in recent years as effective testbeds for conducting research into novel forecasting tools and methods. A challenge of such tournaments involves the distribution of forecasting load across forecasters, since there are often more forecasting questions than an individual forecaster can answer. Intelligent load distribution, or triage, may therefore be helpful in ensuring that all questions have sufficient numbers of forecasts to benefit from crowd-based aggregation and that individual forecasters are matched to the questions for which they are best suited. A possible downside of triage, however, is that it restricts the choices of forecasters, potentially degrading motivation and accuracy. In two studies involving pools of novice forecasters recruited online, we examined the impact of limiting forecaster choice on forecasters’ accuracy and subjective experience, including motivation. In Study 1, we tested the impact of restricted choice by comparing the forecasting accuracy and subjective experience of users who perceived they did or did not have choice in the questions they forecasted. In Study 2, we further tested the impact of restricted choice by providing users with different menu sizes of questions from which to choose. In both studies, we found no evidence that limiting forecaster choice adversely affected forecasting accuracy or subjective experience. This suggests that in large-scale forecasting tournaments, it may be possible to implement choice-limiting triage strategies without sacrificing individual accuracy and motivation.

## Introduction

Accurate prediction of future events is vital for success in most domains, from business ventures to personal life planning to medical decision making. One of the highest stakes domains of prediction is geopolitical forecasting. Geopolitical forecasting is the process of predicting the probability of future geopolitical events across many domains, including international conflict, economic trends, election outcomes, and scientific developments. Policy makers, both public and private, rely on accurate prediction of global events to determine optimal courses of action. One avenue for stimulating research on improving the judgments of human forecasters is to engage researchers in large-scale online geopolitical forecasting tournaments (Tetlock et al., 2014, 2017). The United States Intelligence Advanced Research Projects Activity (IARPA) has organized a series of such forecasting tournaments to promote the development of forecasting methods, Internet-based forecasting systems, and computer-based forecasting tools that can predict world events with improved accuracy over existing best practices. A major challenge in these tournaments is how to efficiently distribute the forecasting load across a large online population of novice forecasters. To guarantee that a sufficient number of well researched unique forecasts are obtained for all questions in the tournament, the labor of forecasting needs to be strategically divided among a finite number of participating forecasters. However, such “triage” assignments must ensure that assignment procedures do not impede the accuracy of individual forecasts or carry an unacceptable cost to user experience in the forecasting system. The current research therefore investigates the impact of procedures that limit choice on both forecast accuracy and on user experiences in a forecasting task with novice forecasters recruited online.

Several factors suggest the necessity of triage systems for online forecasting tournaments. Current best practices for human forecasting (Tetlock and Gardner, 2015; Chang et al., 2016) do not necessarily scale well in a time sensitive tournament where inexperienced forecasters are inundated with novel questions on a weekly basis. The number of forecasting questions in such tournaments is often far greater than the amount for which any individual human forecaster could be expected to supply well-researched predictions, even when supported by computer-based forecasting tools [such as those described in Juvina et al. (2019) and Widmer et al. (2019)]. Additionally, to take advantage of wisdom of crowds effects (Hong and Page, 2004; Surowiecki, 2004; Lee and Danileiko, 2014), multiple independent forecasts are required for each question. The cognitive fatigue from forecasting too many questions is likely to lead forecasters to reduce the variance and independence of individual forecasts (Hirshleifer et al., 2019), reducing the overall quality of forecasts. Triage strategies are therefore necessary to meet these demands.

Perhaps, the most direct triage strategy that can be employed to ensure all questions receive adequate coverage is to restrict forecasters’ choice in which questions they can forecast. That is, rather than permitting forecasters to browse the full list of tournament questions and select only the questions they prefer, only specific questions (such as those most in need of additional forecasts) could be presented to forecasters. Forecasters would then select only from this limited list – or, in the most extreme case, be assigned specific questions with no choice whatsoever. However, implementing a system with this strategy comes with a risk. Restricting a forecaster’s choice to only specific forecasting questions may be detrimental to forecast accuracy. Forecasters may naturally select the questions on topics they are most suited to forecast, due to either domain expertise or interest and motivation. Assuming forecasters have an accurate assessment of their own domain knowledge and are more likely to select questions about which they have greater expertise, forecasters who freely select questions may produce more accurate predictions than forecasters who have restricted question choice. Indeed, recent work has shown that crowds of online participants who freely choose which questions to answer produce more accurate aggregated solutions than crowds of participants who answer assigned questions (Bennett et al., 2018). This finding relies on the assumption that participants have an accurate metacognitive assessment of what they know. This assumption is likely to be met when participants answer general knowledge questions, as was the case in the aforementioned study (Bennett et al., 2018). However, it is not clear if this assumption holds for the types of forecasting questions used in recent large-scale geopolitical forecasting tournaments.

Beyond the specific benefits of choice to general knowledge question performance, having choice generally appears to benefit cognitive performance relative to having no choice. Perceptions of control help regulate effort on cognitive tasks (Schmitz and Skinner, 1993), suggesting that completely eliminating choice may impose motivational and performance costs. However, to the extent that a task is still engaging and enjoyable, the deleterious effects of low control on cognitive tasks may be mitigated (Ruthig et al., 2008). Thus, understanding the effects of restricted choice on subjective experience is as important as understanding direct effects on performance.

However, choice may not necessarily be beneficial to forecasting competitions. Forecasting and other forms of intelligence analysis are information-intensive tasks (Pirolli and Card, 2005) that are much more challenging than typical general knowledge tasks. In a general knowledge task, the answer exists somewhere, and the subjects have to remember it, find it, or deduce it from other pieces of available knowledge. In forecasting tasks, the answers do not exist; they will become known at some point in the future. Thus, a different type of metacognitive assessment is needed – specifically, awareness of what the forecaster is good at predicting or researching, rather than awareness of what the forecaster already knows. Typically, real-world forecasting occurs over an extended time course, during which the world changes and potentially relevant but also irrelevant or misleading evidence accumulates. Due to the novelty and the unparalleled complexity of forecasting tasks, participants may be less accurate in their metacognitive assessments of task difficulty. That is, just because participants have the ability to choose questions to forecast does not mean that they will be able to correctly select the questions they are most likely to forecast accurately, potentially limiting the observed benefits of free choice within forecasting system interfaces. The best forecasters, or “superforecasters” (see Mellers et al., 2015b; Tetlock and Gardner, 2015), are typically skilled at a range of cognitive tasks relevant to forecasting and may exhibit greater metacognitive expertise assessment due to superior ability and experience in forecasting tasks. However, the novice and intermediate level forecasters required to conduct forecasting competitions at scale may lack this metacognitive awareness. For example, in a study employing similar forecasting questions (Summerville et al., 2021, unpublished), we found that participants were not able to distinguish between accurate and inaccurate forecast updates, suggesting that their metacognitive assessment of their own ability might also be limited.

Furthermore, research in behavioral economics (see Thaler and Sunstein, 2008, for a review) shows that decision-makers use different strategies for making choices depending on the size and complexity of the set of available options. When the choice set is small, they tend to engage in thorough research and examine all the attributes of all the options. When the choice set gets large, they tend to use simplifying heuristics to effectively reduce set size. Although freedom of choice is generally desirable, when individuals are faced with a large number of choice options, they tend to delay decision-making, report lower choice satisfaction, and make poorer decisions – an effect known as “choice overload” (Schwartz, 2004). Iyengar and Lepper (2000) also showed that too much choice is detrimental to both performance and satisfaction in decision-making tasks. Additionally, providing choice about irrelevant factors, such as the lighting conditions in a room, may actually impair performance on cognitive tasks, potentially by creating an opportunity for the “wrong” choice on this decision and thus increasing evaluation apprehension on the focal task (Veitch and Gifford, 1996). Thus, it is possible that choice, especially in a large choice set, may actually harm motivation and performance.

Thus, the current research investigated two open questions:

Q1: Does restricting choice influence performance on a forecasting task?

Q2: Does restricting choice affect users’ subjective experience on a forecasting task?

For both questions, the balance of prior research suggested that both directions of effect – choice being helpful or harmful – were equally plausible, and we thus did not have directional hypotheses for either question.

The current studies were run in the context of the latest IARPA forecasting tournament, the hybrid forecasting competition (HFC), which had the specific goal of developing “hybrid” geopolitical forecasting tools that optimally leverage both human and machine inputs. Such tools may include automated recommendation or “triage” systems that utilize machine learning and other strategies to assign questions to forecasters. In the two current studies, we explore the impact of choice restriction on forecasting accuracy using a population of novice forecasters recruited online, similar to (but independent from) the forecaster pool used in the HFC online forecasting tournament.

## Study 1

Study 1 tested the effects of perceived choice on forecasting performance and engagement by directly comparing the performance and experience of users who forecast questions that they self-selected versus users who believed that their question set had been randomly assigned.

### Method

Complete study materials, data, and analysis scripts in R for both studies are available at https://osf.io/rcuk3/?view_only=f9635f5278d14134ac0e459b88e03851. Studies 1 and 2 were run concurrently, but users could participate in only one study. In both studies, in addition to base compensation of $8, participants were informed that the top 10% of forecasters would share a $500 bonus pool as an incentive to make accurate forecasts. Preregistration documents for Study 1 are available *via* https://aspredicted.org/blind.php?x=pv4sm3.

#### Participants

In order to detect a small-to-medium between-conditions effect (*d* = 0.35) with 90% power, we required a minimum of 282 participants. We oversampled to allow for potential exclusions and for a pilot test to estimate completion time to set compensation. Participants were recruited *via* Mechanical Turk. 307 participants (154 male, 149 female, 2 non-binary/nonconforming, and 2 non-disclosed; 239 White, 34 Asian, 31 Black, and 7 Native American; 19 Hispanic/Latino; 287 native English speakers; and 20 non-native or not disclosed fluent speakers) provided usable data; an additional 13 participants who indicated that they were not fluent in English or whose responses provided nonsense or bot-like rationales for forecasts were removed prior to analysis.

#### Materials

The set of 25 forecasting questions in both Studies 1 and 2 was adapted from questions in the IARPA HFC forecasting tournament. In general, these modifications involved changing the dates of a tournament question to ensure that it covered a range beginning after data collection but concluding within 6 weeks. These questions covered topics involving economics (e.g., the closing price of the FTSE 100 stock index), politics (e.g., the monthly approval of the Russian government), and conflict (e.g., the number of civilian deaths in Syria). Questions involved assignment of probabilities to both binary (yes/no) forecasts (“Will the disapproval rate for Japan’s cabinet in NHK’s monthly survey for August be higher than the July rate?”) and continuous numeric bins [“What will be the daily closing price of gold on 15 September 2019? (Less than $1,320; Between $1,320 and $1,400, inclusive; More than $1,400 but less than $1,480; Between $1,480 and $1,560; and More than $1,560)”]. See OSF link for the full set of questions and response bins.

Forecasts were scored using the Brier Score, a common quadratic scoring rule for measuring the accuracy of probabilistic forecasts (Brier, 1950). For ordered multinomial questions involving a continuous range of values (e.g., predicting which of five continuous bins the value of a commodity would fall into), a variant of the Brier Score developed by Jose et al. (2009) was used.

#### Procedure

After providing informed consent, participants were asked to rate their interest in making forecasts about different world regions and topics (e.g., economics and conflict). Next, participants ranked 25 forecasting questions based on which questions they would most versus least like to forecast. All participants then reviewed eight screens of informational material about how to make accurate forecasts.

Participants were then assigned three forecasting questions. Participants in the **choice** condition were told that they would forecast their three top-ranked questions, whereas participants in the **no choice** condition were told that the forecasting questions had been randomly assigned. In reality, all participants were assigned their top three ranked questions. In a debriefing at the end of the study, three participants in the no-choice condition indicated that they recognized these as their top three choices or otherwise indicated some suspicion that the assignment had not been random. Consistent with our preregistration, these participants were excluded from analysis.

After making forecasts, participants provided a brief summary of the rationale for their forecast for each question. They then rated the ease versus difficulty of the task, their enjoyment, their confidence, their motivation to make accurate forecasts, and their prior knowledge of the forecasting topics prior to beginning the study. They then provided demographic information and were debriefed.

### Results

**Figure 1:**
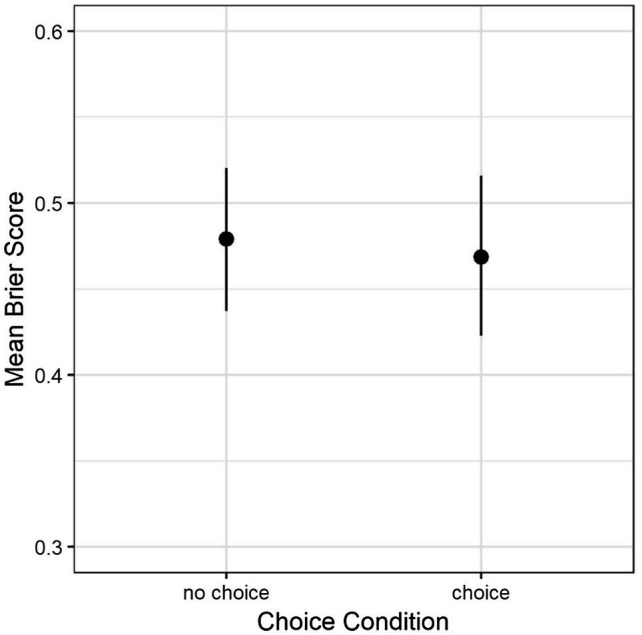
Mean accuracy (Brier score) by condition. Error bars are 95% C.I. Note that more accurate forecasts have lower scores.

The primary question in this study was whether perceiving that forecasting questions were chosen versus assigned would affect forecast accuracy. An independent samples t-test indicated no such difference, *t* (295) = 0.47, *p* = 0.64, and *d* = 0.05 (see Figure 1).

We next examined whether participant subjective experiences differed between conditions. The conditions did not differ in their ratings of the ease of the task (*p* = 0.81), their enjoyment of the task (*p* = 0.38), their confidence in the accuracy of their forecasts (*p* = 0.96), or their motivation to forecast accurately (*p* = 0.43). Moreover, the groups did not differ in their prior knowledge of topics (*p* = 0.99). See Table 1 for means.

**Table 1:** Mean user ratings by condition.

Condition	Difficulty of task	Enjoyment of task	Confidence in forecasts	Motivation to be accurate	Prior knowledge of topics
No choice	3.43	3.66	3.15	4.53	2.12
Choice	3.40	3.77	3.14	4.47	2.12

### Discussion

Although Study 1 found no evidence that perception of choice restriction impacted forecast accuracy, this conclusion is hampered in two main respects. First, participants all forecasted their most desired questions, meaning that performance may have been maximal. To the extent that an effect is driven by a benefit of choice (e.g., due to participant metacognition about accuracy or to a boost in motivation and effort), this study would not have detected the difference. Second, the manipulation of choice versus non-choice was fairly subtle. To address both of these concerns, Study 2 used a more explicit manipulation of choice set *via* the random assignment of menu sizes and sets.

## Study 2

### Method

Preregistration documents for Study 2 are available *via* https://aspredicted.org/blind.php?x=4r68h4.

#### Participants

We followed the recommendations of Lakens and Evers (2014) for a minimum of 50 participants per between-subjects cell and oversampled to allow for potential exclusions or missing data. Participants were recruited *via* Mechanical Turk. 270 participants (130 male, 130 female, 1 non-binary/nonconforming, and 9 non-disclosed; 214 White, 29 Black, 18 Asian, and 7 Native American; 23 Hispanic/Latino; 254 native English speakers; and 7 non-native fluent speakers) provided usable data. Additionally, 56 participants began the study but withdrew before making forecasts; seven responses that provided nonsense or bot-like rationales for forecasts were removed prior to analysis. The obtained sample thus allowed us to detect a medium effect (Cohen’s *f* = 0.20) with 80% power.

#### Procedure

After providing informed consent, participants were asked to rate their interest in making forecasts about different world regions and topics (e.g., economics and conflict) Next, participants ranked the same 25 forecasting questions used in Study 1 based on those they would most versus least like to forecast. This ranking was framed as a general interest in their forecasting preferences, and participants were told that their ranking would not be used to assign forecasting questions in the main task. Participants then reviewed eight screens of informational material about how to make effective forecasts.

Participants were randomly assigned to one of five menu size conditions: 3, 5, 10, 15, and 25 options. Participants in the 25-option condition saw a menu of all 25 forecasting questions in random order. Participants in the 5-, 10-, and 15-option conditions saw a menu consisting of a randomly generated subset of forecasting questions (5, 10, and 15, respectively), presented in a randomized order. Participants in the 3-option condition were taken directly to the forecasting page and told they had been assigned the three questions on the page. All other participants were first presented with the menu and told to select three questions to forecast; the survey platform required that they select three questions to advance to the forecasting page.

All participants then submitted forecasts for the three questions they had selected. After making forecasts, participants provided a brief summary of the rationale for their forecast. They then rated the ease versus difficulty of the task, their enjoyment, their confidence, their motivation to make accurate forecasts, and their prior knowledge of the forecasting topic prior to beginning the study. They then provided demographic information and were debriefed.

### Results and Discussion

**Figure 2:**
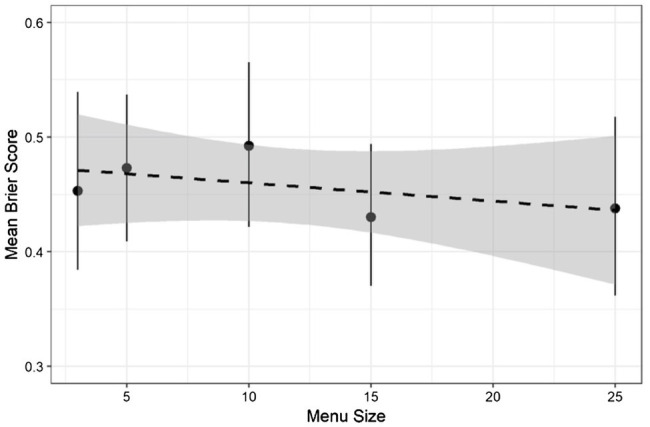
Mean accuracy by condition. Error bars are 95% C.I. Regression line is shown as a dashed line with a 95% C.I.

To determine if menu size impacted forecast accuracy, accuracy scores were first submitted to a one-way ANOVA. This revealed no difference between conditions, *F* (4, 265) = 0.54, *p* = 0.71, and generalized *η*^2^ = 0.008. Additionally, a linear regression of accuracy on menu size as a continuous variable indicated that there was no linear trend, *t* (268) = 0.77, *p* = 0.44, and *η*^2^ = 0.002 (see Figure 2).

As in Study 1, we next examined participant experience using a one-way ANOVA. As in Study 1, there was no difference between conditions for ratings of the ease of the task (*p* = 0.93), users’ confidence in the accuracy of their forecasts (*p* = 0.96), or in their reported motivation to forecast accuracy (*p* = 0.88). These effects remained non-significant when regressed on menu size as a continuous predictor. However, there was some evidence that participants’ enjoyment of the task did differ between conditions, in an ANOVA: *F* (4, 256) = 1.63, *p* = 0.17, and *η*^2^ = 0.02; in a linear regression: *t* (259) = 1.74, *p* = 0.08, and *η*^2^ = 0.01. Post-hoc contrasts revealed that enjoyment differed significantly between the menu sizes of 3 vs. 15: *p* = 0.015. Additionally, menu sizes predicted ratings of prior knowledge in an ANOVA: *F* (4, 256) = 3.68, *p* = 0.006, and *η*^2^ = 0.05; in a linear regression: *t* (259) = 2.97, *p* = 0.003, and *η*^2^ = 0.03. *Post-hoc* contrasts revealed that users who were assigned the three questions that they forecasted indicated that they had less knowledge of the topics than users who had selected from menus of 10 [*t* (256) = 3.03, *p* = 0.03], 15 [*t* (256) = 2.09, *p* = 0.04], or 25 [*t* (256) = 3.02, *p* = 0.003] and that users who selected from a menu of 5 felt they had less knowledge than users who selected from menus of 10 [*t* (256) = 2.27, *p* = 0.02] or 25 [*t* (256) = 2.27, *p* = 0.02]. No other contrasts were significant (*p* > 0.19) (see Table 2).

**Table 2:** Mean user ratings by condition.

Menu size	Difficulty of task	Enjoyment of task	Confidence in forecasts	Motivation to be accurate	Prior knowledge of topics
3	3.55	3.47	3.11	4.55	1.55
5	3.36	3.71	3.22	4.55	1.71
10	3.45	3.80	3.15	4.44	2.11
15	3.48	4.04	3.12	4.48	1.94
25	3.50	3.87	3.21	4.46	2.12

Thus, Study 2 found no evidence that offering forecasters different sized menus of questions to forecast impacted forecast accuracy. Importantly, this includes the condition with menu size 3, in which participants genuinely had no choice or control over the questions they forecasted (as they were required to forecast three out of three available questions).

## General Discussion

Both Study 1 and Study 2 found no evidence that restricting forecaster choice impacted forecast accuracy. Neither restricting perception of choice (Study 1) or reducing the size of the question set that forecasters could select from (Study 2) significantly reduced the accuracy of participants’ predictions. Importantly, this was also true in the smallest menu size (3) condition of Study 2, in which participants genuinely had no choice in which questions to forecast (i.e., a “hard assignment” triage strategy). Similarly, no difference was found in participants’ perception of the ease of forecasting the questions or in their motivation to make accurate forecasts in either choice restriction manipulation, although participants who were offered larger menu sizes in Study 2 reported enjoying the task more. This study therefore suggests that online forecasting systems can impose assignment procedures without clear costs to user performance or engagement, though offering users even limited choice did seem to benefit user experience relative to a strict assignment procedure.

One obvious concern in the current data is that this conclusion is largely driven by null effects for accuracy. Although the studies offer adequate power to detect medium effects, it is possible that there are weak differences that went undetected. These results are thus best interpreted as suggestive evidence about the degree of methodological flexibility available in design choices for forecasting interfaces, rather than an absolute conclusion that such choices will never have costs to accuracy or engagement.

This caveat is also important given the context of the current study. In contrast to lengthy forecasting tournaments in which maintaining user engagement over the course of months is critical, the current research involved novice forecasters in a single, hour-long session. It is therefore possible that differences in motivation might emerge over a longer time span or that the small differences in enjoyment found in Study 2 could compound to have non-trivial effects on retention. It is also possible that large forecasting tournaments attract and retain participants who enjoy doing research and generating forecasts for a variety of questions and challenge levels. Their interests might vary from day to day and might go significantly beyond their expertise. For example, according to Mellers et al. (2015a), participants who are actively open-minded, intellectually curious, and have a growth mindset tend to generate more accurate forecasts. For this kind of participants, restricting choice could undermine some of the incentives to participate in forecasting tournaments.

Another possibility is that the participants in the current studies lacked domain knowledge for any of the questions included, and thus the lack of effect of choice restriction on accuracy is due to participants being equally suited to forecasting all questions. However, the current research drew from the same broad population as the larger forecasting tournament (i.e., Amazon Mechanical Turk workers). Although users who choose to participate in a months-long forecasting tournament may have somewhat greater expertise than the general user in this population, the fact that they come from the same underlying pool mitigates this risk somewhat. This suggests that restricting question choice in the tournament would likely also not negatively impact forecasting accuracy.

While we did not observe a cost to restricting forecaster choice in the current studies, a remaining question is to identify the optimal method for determining which questions to present to which forecasters. If the observation of Bennett et al. (2018) that voluntary answers produce more accurate crowds than mandatory answers to general knowledge questions is due to individuals possessing accurate metacognition about their domain expertise and appropriately selecting questions rather than an effect of choice versus assignment; then, it may be possible to cultivate “wiser” crowds of forecasters with intelligent question assignment. While the population used in our study may not tend to possess sufficient metacognition about forecasting domain expertise to consistently select the questions they are most suited to forecasting, forecasters assigned only questions which they are well suited to forecast should produce forecasts as accurate (if not better) than forecasters with sufficient metacognitive awareness who can freely select questions.

One approach would be to assign to each forecaster question sets that cover a broad range of topics early in the tournament to observe any domain expertise present in each forecaster. Performance data on these early questions could then be used to refine future questions sets assigned to each forecaster to attempt to aggregate over a select crowd (see Mannes et al., 2014) for each question that may have better accuracy than if questions are entirely randomly assigned to forecasters. That is, forecasters would be shown questions they are likely to perform better on, while also balancing forecasters across questions based on dispositional factors associated with forecasting success (see Mellers et al., 2015a), such that all questions are covered by equally skilled forecasters overall. Forecasters could also on occasion be assigned questions outside their observed expertise to either validate that their true expertise has been identified or recalibrate to a different topic that better matches their real expertise. Some degree of choice could also be allowed within the early questions sets to detect the type of questions each forecaster prefers to forecast. Accuracy data on these questions could then be compared to question selection strategies to determine the degree of metacognitive awareness each forecaster has of their forecasting expertise. Forecasters with more metacognitive awareness (such as those similar to superforecasters) could then be offered a greater degree of choice in question selection, while restricting the choice of users less able to successfully self-select questions to questions on which they are empirically likely to perform better.

The work presented here relies on the assumption that triaging is a necessary and beneficial procedure. It would be useful for future research to establish a task load (e.g., number of forecasting questions) at which performance and subjective experience clearly start to deteriorate and apply the amount of triage that is most likely to be beneficial to performance and engagement.

While the limited nature of the current studies must be taken into account, the results suggest that restricting forecaster choice in the context of a large-scale Internet-based forecasting tournament may viably be implemented without significantly sacrificing forecasting accuracy.

## Data Availability Statement

The datasets presented in this study can be found in online repositories. The names of the repository/repositories and accession number(s) can be found at: https://osf.io/rcuk3/?view_only=f9635f5278d14134ac0e459b88e03851.

## Ethics Statement

The studies involving human participants were reviewed and approved by the New England Independent Review Board. The patients/participants provided their written informed consent to participate in this study.

## Author Contributions

CW, AS, IJ, and BM designed the studies and contributed to writing the manuscript. CW collected the data primarily. AS analyzed the data primarily. All authors contributed to the article and approved the submitted version.

### Conflict of Interest

CW, AS, and BM were employed by the company Kairos Research.

The remaining author declares that the research was conducted in the absence of any commercial or financial relationships that could be construed as a potential conflict of interest.
